# RTM Production Monitoring of the A380 Hinge Arm Droop Nose Mechanism: A Multi-Sensor Approach

**DOI:** 10.3390/s16060866

**Published:** 2016-06-14

**Authors:** Gabriele Chiesura, Alfredo Lamberti, Yang Yang, Geert Luyckx, Wim Van Paepegem, Steve Vanlanduit, Jan Vanfleteren, Joris Degrieck

**Affiliations:** 1 Department of Material Science and Engineering, Ghent University, Technologiepark 903, 9052 Zwijnaarde, Belgium; geert.luyckx@ugent.be (G.L.); wim.vanpaepegem@ugent.be (W.V.P.); Joris.Degrieck@ugent.be (J.D.); 2 Department of Mechanical Engineering, Vrije Universiteit Brussel (VUB), Pleinlaan 2, 1050 Elsene, Belgium; allamber@vub.ac.be (A.L.); Steve.Vanlanduit@uantwerpen.be (S.V.); 3 Centre for Microsystems Technology (CMST), IMEC and Ghent University, Technologiepark 15, 9052 Zwijnaarde, Belgium; yang.yang@ugent.be (Y.Y.); Jan.vanfleteren@ugent.be (J.V.); 4 Faculty of Applied Engineering, University of Antwerp, Salesianenlaan 90, 2660 Hoboken, Belgium

**Keywords:** fibre optics, dielectric analysis, composite materials, production monitoring

## Abstract

This research presents a case study of production monitoring on an aerospace composite component: the hinge arm of the droop nose mechanism on the Airbus A380 wing leading edge. A sensor network composed of Fibre Bragg Gratings, capacitive sensors for cure monitoring and thermocouples was embedded in its fibre reinforced lay-up and measurements were acquired throughout its Resin Transfer Moulding production process. Two main challenges had to be overcome: first, the integration of the sensor lines in the existing Resin Transfer Moulding mould without modifying it; second, the demoulding of the component without damaging the sensor lines. The proposed embedding solution has proved successful. The wavelength shifts of the Fibre Bragg Gratings were observed from the initial production stages, over the resin injection, the complete curing of the resin and the cooling-down prior to demoulding. The sensors proved to be sensitive to detecting the resin flow front, vacuum and pressure increase into the mould and the temperature increase caused by the resin curing. Measurements were also acquired during the post-curing cycle. Residual strains during all steps of the process were derived from the sensors’ wavelength shift, showing values up to 0.2% in compression. Moreover, the capacitive sensors were able to follow-up the curing degree during the production process. The sensors proved able to detect the resin flow front, whereas thermocouples could not measure an appreciable increase of temperature due to the fact that the resin had the same temperature as the mould.

## 1. Introduction

The composite industry is broadening its applications to more and more markets, as a strong confidence in these materials has generally been achieved nowadays. Two relevant examples of markets in which composites have seen a growth over the last decade are the aerospace and the automotive industry. Demand for composite materials in the U.S. aerospace market grew by 10.2% in 2013 and analysts expect a growth of between 8% and 13% over the coming years [1]. A similar scenario is foreseen for the automotive industry where, however, the limiting factors towards mass production are represented by the high cost of raw carbon fibre materials and long manufacturing times. The Resin Transfer Moulding (RTM) production process is widely used in these applications and likewise it has seen a growth of about 5% in 2013 [2]. The possibility of creating net-shape components of complex geometry and the advantage of having greater process parameter variability make this process more preferable than others. Often, a decisive factor in these closed-mould applications is the cycle time which has to be as short as possible in order to maximize the production output. However, this strong requirement leads in some cases to wrong parameter selection, as it could lead to an excessive curing temperature or too fast a cooling-down rate. Consequently, the final quality of the produced part might be jeopardized, as for instance induced thermal stresses could lead to premature cracks, or too short a curing cycle might leave some uncured resin areas, which will inevitably require scrapping the composite part. A process optimization study should be considered in order to avoid such situations.

Composite materials offer, amongst others, the advantage of embedding inside the stack of plies sensors which can then be used for Structural Health Monitoring purposes [3,4,5] and more widely for monitoring the whole composite life cycle. In other words sensors can be exploited in the early stages for the production monitoring, in order to gain information on process parameters, thus avoiding the aforementioned drawbacks. In industry, common practice for large production series or high-end applications, is characterizing the resin curing cycle on a reference sample using Differential Scanning Calorimetry (DSC) [6]. This technique allows defining the glass transition temperature *T_g_* [°C] of the polymer and its degree of cure curve by measuring its heat flow during a defined curing profile (*i.e.*, usually isothermal). However, due to limitations of the equipment itself, only small samples of material can be characterized (2 mm in diameter and less than 100 mg in mass), limiting the applicability of such results to full scale components, where resin content might vary significantly depending on geometry or temperature variations. Another experimental technique, known as Dielectric Analysis (DEA), is spreading across the industry for its advantage of using disposable sensors which can be embedded inside the material to be characterized, thus allowing for *in situ* monitoring of real composite components. When the application requires it, these sensors can also be integrated in the mould surface and be reused for several hundreds of cycles, in a cost effective way throughout the process chain. Residual strains, instead, can be quantified by means of embedded fibre optic sensors which capture the resin shrinkage within two adjacent composite plies arising during the production process. Generally higher curing temperatures allow for shorter cycle times and higher *T_g_*, but also involve larger thermal stresses.

Several studies on process monitoring of composite materials using different types of sensors can be found in the literature. For instance Kikuchi explored several types of electrical and optical sensors (both surface mounted and embedded) for the purpose of monitoring resin moulding processes [7,8]. More specifically Yildiz *et al.* used both Fibre Bragg Gratings (FBGs) and Edge Fibres (EFs) to monitor the resin flow front inside a closed mould. They followed the curing process by means of Fresnel Reflection Refractometry (FRR) sensors, a special type of fibre optics with a cleaved end-facet capable of detecting the refractive index change of the surrounding resin material. Measuring the light intensity variation throughout the curing process allows retrieving the resin curing degree curve [9]. Although this sensor is very small in dimensions and can easily be placed between two composite plies (*i.e.*, very limited layer distortion), it does not allow multiplexing (*i.e.*, only one sensor per optical line is allowed). Dunkers has used specifically developed long-period gratings with a fluorescent coating to monitor the flow of a polymer inside a mould along the whole fibre length [10]. The equipment he has used required a very costly optical spectral analyser unit and this represents for most industrial production processes a strong limitation to its applicability. Nevertheless, these different types of optical sensors were meant to be used as disposable. Keulen *et al.* partially solved this problem by designing a mould with surface integrated EF sensors, so that they could be reused [11]. However, up-scaling the single sensor into a sensor network, capable of measuring the curing over the whole mould area becomes challenging and also costly. On the contrary electric sensors normally used in DEA, are relatively cheap to manufacture and can therefore be disposable if embedded in a network or, in a more costly configuration, integrated into the mould. Danisman *et al.* have developed a point-voltage sensor network which was integrated inside a planar rectangular mould and he proved that such a point-sensor is capable of capturing the resin flow even for fast injection processes [12]. Among all kinds of electrical sensors, capacitive sensors are more widely used for cure monitoring, since the change in permittivity of the resin during its curing process can be captured by measuring the capacitance variation. Hardis *et al.* compared different curing monitoring techniques in an epoxy resin: DEA proved to agree well with the results obtained by the generally accepted standard DSC technique [13]. Yenilmez designed a capacitive sensor grid and integrated it into a RTM plane mould; he could monitor the filling of the mould on its full area and detect the degree of cure based on his sensing grid [14]. More recently, O’Dwyer *et al.* estimated the total residual strain measured from fibre optic sensors which were embedded in a two layers carbon fibre prepreg coupon and related it to the cure degree profile obtained by dielectric sensors [15]. In a previous research activity the authors have embedded a sensor network based on FBGs in a carbon fibre reinforced preform produced by RTM [16]. The wavelength shifts of the sensors were recorded over the entire production process. However, due to the missing temperature information throughout the process, no quantitative residual strains could be derived.

**Figure 1:**
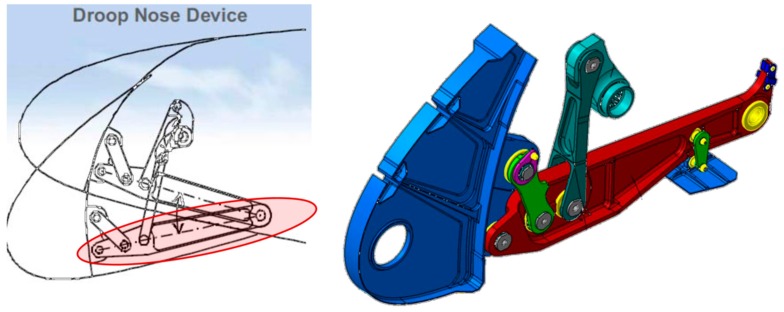
Schematics of the hinge arm of the droop nose mechanism on the Airbus A380 wing leading edge (side section) [17,18].

In the current work, a multi-sensor approach, which combines dielectric sensors, temperature sensors and strain sensors, has been used to monitor all the curing process steps (*i.e.*, resin injection, curing, demoulding and post-curing) of a glass fibre composite component produced by RTM featuring the hinge arm of the droop nose mechanism on the Airbus A380 wing leading edge (Figure 1). The component was the final result of a previous research project, the European Aircraft Integrated Structural Health Assessment II (AISHA II) project, where the feasibility of translating the design and production of a conventional metal component into a composite one was proved. Since the aim of this paper is to present results on the cure monitoring of this component and not to describe its design process, the reader is invited to consult references to the project for more details [17]. For the same reason, a different resin system and reinforcement fibres were chosen instead of the ones selected in AISHA II. It is worth to mention that not all sensors can be safely embedded in a composite structure which is intended to be employed during normal operation. In fact, large sensors can act as damage initiators and therefore they should be used only during preproduction in order to gain information on the manufacturing process. In this sense the multi-sensor approach is only foreseen for production monitoring.

In the hinge arm preform, a fibre optic line carrying five FBG sensors, together with two capacitive sensors and two thermocouples were embedded in the composite stacking. While the fibre optics and the thermocouples were commercially available sensors, the capacitive sensors were developed in-house. Their printed circuit board-based technology allowed us to design relatively low-cost sensors, meant to be disposable, yet strong enough to be integrated in industrial production. The part was produced by the RTM process, then demoulded and post-cured in an oven. The sensor readouts were acquired throughout the whole curing and post-curing process. The paper is structured as follows: firstly, the working principles of FBGs and capacitive sensors for cure monitoring will be introduced. A formulation to derive residual strains and degree of cure from the measured data will be given. Secondly, the experimental setup and its associated procedure will be explained. Thirdly, the result section will present the major findings from the cure monitoring experiment. Lastly, some conclusions on the achievements of this research and a perspective on some future work will be given.

## 2. FBG Sensors for Residual Strain Measurements

### 2.1. Basics on FBGs

**Figure 2:**
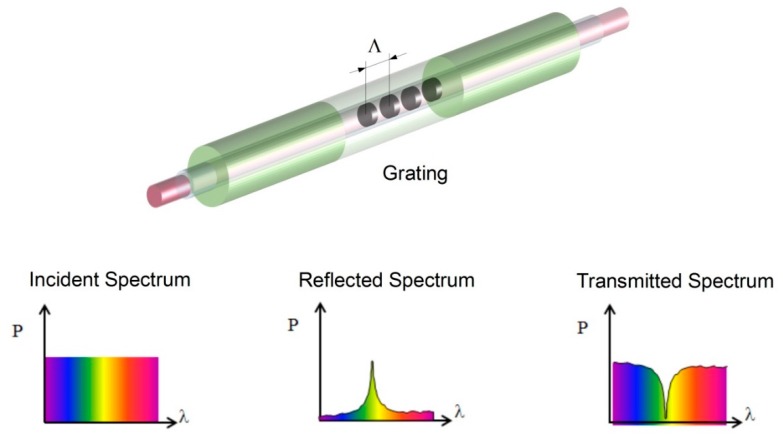
Fibre Bragg grating schematic representation.

Optical fibre Bragg gratings are point sensors where by means of an interfering UV light pattern a periodic variation of the refractive index is induced (*i.e.*, the Bragg grating) to the fibre core. When broadband light is sent through the fibre core, the grating acts as a selective filter and reflects back part of the initial spectrum (Figure 2).

The reflected spectrum is characterized by its Bragg wavelength λ_B_ via the following equation [19,20]:

(1) $$\lambda_{B}=2n_{eff}\Lambda$$

where *n_eff_* is the effective refractive index of the grating and Λ is the grating period. Typical Bragg wavelengths are in the range of 830, 1300 or 1550 nm, depending on the grating inscription technology used and the fibre optic core refractive index. The great advantage of this technology lays in the fact that the grating period can be tuned in such a way to have a specific Bragg wavelength, hence allowing inscribing multiple gratings on the same fibre optic line and reading them out on a single channel. An arbitrary variation of strain and temperature applied on the grating will induce a shift on the Bragg wavelength, according to Equation (2) [21]:

(2) $$\frac{{\Delta \lambda }_{B}}{\lambda_{B}}=\;\left(\frac{1}{n_{eff}}\frac{dn_{eff}}{dT}+\frac{1}{\Lambda }\frac{d\Lambda }{dT}\right)\Delta T+\left(\frac{1}{n_{eff}}\frac{dn_{eff}}{d\varepsilon }+\frac{1}{\Lambda }\frac{d\Lambda }{d\varepsilon }\right)\Delta \varepsilon$$

The first term on the right hand side of Equation (2) represents the Bragg-wavelength shift induced by a temperature variation Δ*T* which is the contribution of the thermo-optic coefficient and the thermal expansion coefficient. Typical values of the thermo-optic coefficient ξ and of the thermal expansion coefficient α*_f_* for FBG sensors with germanium (Ge0_2_) doped silica core optical fibre are respectively 5.95 × 10^−6^ K^−1^ and 0.55 × 10^−6^ K^−1^ [21,22]. The FBG thermal sensitivity *K_T_* for a 1550 nm Bragg wavelength in the temperature range 20–100 °C becomes therefore [23]:

(3) $$K_{T}=\frac{{\Delta \lambda }_{B}}{\lambda_{B}\Delta T}=\;\left(\frac{1}{n_{eff}}\frac{dn_{eff}}{dT}+\frac{1}{\Lambda }\frac{d\Lambda }{dT}\right)=\xi +\alpha_{f}≅6.5\;\times 10^{-6}\;K^{-1}$$

Similarly, the second term represents the contribution of a strain variation Δε to the Bragg wavelength shift, which is given by a physical change in the grating periodicity and by a strain-optic induced change in the refractive index. The strain sensitivity *K*_ε_ can be rewritten as:

(4) $$K_{\varepsilon }=\frac{\Delta \lambda_{B}}{\lambda_{B}\Delta \varepsilon }=\;\left(1-p_{e}\right)≅7.8\times 10^{-7}\mu \varepsilon^{-1}$$

where a typical value of the photo-elastic coefficient for bulk silica fibre is *p_e_* ≈ 0.22 [20,24].

### 2.2. Temperature Compensation

As it has been shown from Equation (2) in Section 2.1, the FBG wavelength shift is dependent on both strain and temperature effects, therefore when only the strain contribution is desired, knowledge of temperature is required. This can be achieved in several ways, for example by having a second strain-free FBG close to the principal FBG or by having a thermocouple close to the FBG [25]. Pal *et al.* proposed a temperature compensation method based on a polynomial function in the form:

(5) $$\frac{{\Delta \lambda }_{B}}{\lambda_{B,ref}}=a\;\Delta T+b\;\Delta T^{2}$$

where *a* and *b* are coefficients defined by a temperature calibration test, Δ*T* is the temperature difference with respect to a reference temperature *T_ref_* and λ*_ref_* its corresponding reference wavelength [26]. In the case of a germanium (Ge0_2_) doped silica core FBG the temperature coefficients are respectively *a* = 6.33 × 10^−6^ K^−1^ and *b* = 7 × 10^−9^ K^−2^ [22].

### 2.3. Residual Strain Measurements

When considering the case of a FBG embedded in a composite material which is subjected to an arbitrary strain variation, one should account for the strain transferred from the composite material to the sensor. The problem has been widely addressed in the literature and for the purpose of this paper a distinction has to be made between the case of a uniaxial strain applied in the fibre direction and the case of a general multi-axial strain. In the first case, the response of the sensor is a neat peak which might shift after embedding but maintains its shape, while in the second case the reflected spectrum results in a peak splitting after embedding. This is highly dependent on the composite reinforcement architecture and on the manufacturing process used [22]. However, in the case of simple reinforcement architecture such as the case of an optical fibre embedded along the direction of a unidirectional (UD) reinforcement and in a production process which does not involve high applied pressure, the chances of having a distorted spectrum are limited. Moreover a coated FBG is far less sensitive to transverse strain than a stripped fibre, since the coating acts as a buffer which filters transverse effects [27,28]. In this work coated fibres were embedded along the fibre reinforcing direction or in such manner to diminish the transverse strain effect. In the following, it will hence be referred to the case of a uniaxial strain applied on a coated fibre. During the curing process of a composite material chemical shrinkage is induced, once that the liquid resin starts to solidify binding the fibres together. Along with this phenomenon, a temperature increase (*i.e.*, exothermal reaction) is typically induced by the resin chemical reaction. Due to the effect that the composite exerts on the sensor, new temperature and strain sensitivities $K_{T}^{\prime }$ and$\;K_{\varepsilon }^{\prime }\;$need to be considered. The strain sensitivity for an embedded fibre is similar to the one of a bare fibre, while the temperature sensitivity of an embedded fibre is lower and can be considered equal to its thermo-optic coefficient [29,30,31]. Equation (2) can thus be rewritten as:

(6) $$\frac{{\Delta \lambda }_{B}}{\lambda_{B}}=K_{T}^{\prime }\Delta T\;+\;K_{\varepsilon }^{\prime }\left(\varepsilon_{app}+\varepsilon_{th}\right)≅\xi \Delta T+K_{\varepsilon }\varepsilon_{tot}$$

where ε*_app_* and ε*_th_* are the applied strain and the thermal strain on the fibre surroundings and they can be considered as total strain read by the FBG. If one isolates the term ε*_tot_* in Equation (6), the residual strain induced by the resin curing process can be evaluated.

## 3. Use of Capacitive Sensors for Cure Monitoring

As mentioned in Section 2.3, during the curing, the physical and chemical properties of the polymer undergo a change. Particularly, the change in dielectric properties can be monitored by means of DEA in order to define the curing evolution. A capacitive sensor is placed directly in contact with the resin and an AC current, sweeping in frequency, is applied [13,32].

The resin medium is characterized by a lossy component contributed by the dipole relaxation and the migrating ions, and by a capacitive component given from the dipoles existing in the material. In the absence of any applied electric field the ions and dipoles are randomly oriented. When an alternating electric field is induced, the dipoles will try to orient according to the direction of the electromagnetic force field. Since the reorientation of the dipoles, when the electric field changes, is not immediate, a delay between the applied voltage and the induced current will occur. By measuring the change in amplitude and phase shift of the return signal, the complex permittivity of the material ε* can be measured; the latter is defined as:

(7) $$\varepsilon^{*}=\varepsilon^{\prime }-i\varepsilon^{\prime \prime }$$

with ε*ꞌ* the permittivity of the material and ε*ꞌꞌ* the dielectric loss. The dissipation factor *DF* can be defined as the ratio between the dielectric loss ε*ꞌꞌ* and the permittivity ε*ꞌ*, which is also equal to the tangent of its loss angle δ:

(8) $$DF=\frac{\varepsilon \prime \prime }{\varepsilon^{\prime }}=tan\;\delta$$

These three defined parameters describe the polarization of a dielectric material by dipole orientation in an applied electric field. However, in addition to the dipole orientation phenomenon, a free-ion migration is also taking place concurrently. The loss factor can therefore be expressed by the contribution to the loss factor of the ions migration $\varepsilon_{ion}^{\prime \prime }$ and of the dipoles oscillation $\varepsilon_{dipole}^{\prime \prime }$ [33]:

(9) $$\varepsilon^{\prime \prime }=\varepsilon_{ion}^{\prime \prime }+\varepsilon_{dipole}^{\prime \prime }$$

The conductive component $\varepsilon_{ion}^{\prime \prime }$ can be expressed by:

(10) $$\varepsilon_{ion}^{\prime \prime }=\;\frac{\sigma }{2\pi f\varepsilon_{0}}$$

where σ [S/m] is the ion conductivity, *f* [Hz] the frequency at which the signal is acquired and ε*_0_* [F/m] is the permittivity of vacuum (8.85 × 10^−12^ F/m). At the early curing stage, the resin viscosity is very low and the ions freely moving into the medium can result in a significant increase of ion conductivity σ. When low frequencies are considered, this contribution can become the predominant one and the dipole reorientation effect $\varepsilon_{dipole}^{\prime \prime }$ can therefore be neglected:

(11) $$\varepsilon_{ion}^{\prime \prime }\gg \;\varepsilon_{dipole}^{\prime \prime }\;\to \;\varepsilon^{\prime \prime }=\frac{\sigma }{2\pi f\varepsilon_{0}}$$

Some attention should also be paid to possible polarization effect which can occur at the sensor electrodes, where free-ions accumulate and could result in distorting the measurements [34]. In addition, the ion conductivity σ, which is as previously stated directly related to the ions mobility, can be related to the ion viscosity [Ω/m] through the equation:

(12) $$ion\;viscosity=\;\frac{1}{\sigma }$$

The ion viscosity is physically related to the resin viscosity and therefore an idea on the curing evolution can already be obtained when looking at the ion viscosity curve. As the curing evolves, the mobility of the ions and the rotation of the dipoles become more and more limited. This results into decreasing ion mobility and a relaxation of the dipoles. In addition, the curing evolution can also be described by the function [13]:

(13) $$\alpha \left(t\right)=\;\frac{log\left(\varepsilon_{min}^{\prime \prime }\right)-log\left(\varepsilon_{t}^{\prime \prime }\right)}{log\left(\varepsilon_{min}^{\prime \prime }\right)-log\left(\varepsilon_{\infty }^{\prime \prime }\right)}$$

where α is the percentage of cure at time *t*, $\varepsilon_{min}^{\prime \prime }$ is the loss factor measured at the minimum ion viscosity (max ion conductivity), $\varepsilon_{t}^{\prime \prime }\;$is the loss factor at time *t* and $\varepsilon_{\infty }^{\prime \prime }$ is the loss factor at the end of curing.

## 4. Experimental Setup

### 4.1. RTM Setup

**Figure 3:**
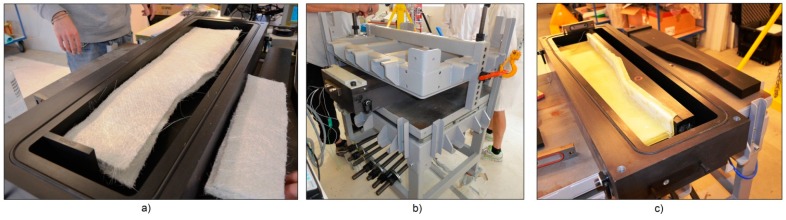
RTM production process of the A380 hinge arm: lower half-mould during preform preparation (**a**); clamping frame used to seal the mould (**b**) and hinge arm demoulding (**c**).

The A380 hinge arm was the final result of the European AISHA II project, which was intended to prove the feasibility of translating the design of an existing metal component into a composite one [17]. The component was made via the RTM process, by means of an alternating pump which was used to mix the epoxy resin and inject it into the mould. The latter was made of a lower part and an upper lid closed and sealed together with the inner composite preform being impregnated by the flowing resin. In Figure 3 an overall view of the mould and of the component during the production stages is presented. In order to obtain the vacuum and pressure tightness, a clamping frame—Figure 3b—was used to apply a contracting mechanical pressure on the mould lid. The mould had an inlet at one extremity and an outlet on its diagonally opposite end.

After demoulding, the hinge arm needed some post-production milling operations to drill the pin holes and trim it to its final shape contour. In addition, the aluminum mould was not equipped with heating/cooling system and therefore the process was performed at ambient temperature.

### 4.2. Strain Monitoring

The strains have been derived from the FBGs wavelength peak shift throughout the curing and the post-curing processes. For the RTM curing process of the A380 hinge arm a Micron Optics^®^ (Atlanta, GA, USA) static interrogator sm125–500 with 1510–1590 nm bandwidth and a wavelength repeatability of ±0.2 pm was used. For the oven post-curing a FBG-Scan804 interrogator from FBGS^®^ Technologies GmbH (Jena, Germany) was used, instead. The latter had a 1510–1590 nm bandwidth and a wavelength precision of ±1 pm. The optical fibre sensor lines were DTG^®^ from FBGS^®^ Technologies GmbH having inscribed FBGs in the range of 1510–1590 nm [22]. The cladding diameter was 125 μm and the fibres were coated with an organic modified ceramic (Ormocer^®^) material with an overall outer diameter of 195 μm.

### 4.3. Cure Monitoring

DEA measurements were acquired during the RTM curing and following the oven post-curing process. Two different types of planar inter-digit sensors were used, namely an in-house developed sensor and a commercially available IDEX 115 provided by Netzsch GmbH (Selb, Germany). Capacitance and dissipation factor measurements were performed on two different pieces of equipment as well. The in-house developed sensor was connected to an HP 4284A Precision LCR (Hewlett-Packard LTD, Hyogo, Japan) and measurements were performed with frequency sweep ranging from 100 Hz to 1 MHz with 10 points per decade on an in-house developed planar inter-digit capacitive sensor. The time required per sweep was approximately 16 s. The other sensor was connected to a dielectric analyser DEA 288 also from Netzsch GmbH. In this case the measurements were done with a frequency sweep ranging from 1 Hz to 1 MHz. The benefit of having two different pieces of equipment was of validating the results obtained from the in-house developed sensor. For this purpose, the high-precision LCR meter was used on the in-house developed sensors in order to obtain more accurate measurements. Temperature values were acquired with the DEA 288 using two type K thermocouples which were installed next to the capacitive and the FBG sensors. The LCR meter’s raw data were further post-processed via Matlab^®^, while for the DEA 288 the data were visualized via the Proteous^®^ analysis software provided by Netzsch.

## 5. Sensors Embedding

The A380 hinge arm was intended to be originally made of a carbon fibre reinforcement preform for design requirements (*i.e.*, higher stiffness and strength to failure). However, for the current research purpose, and given the higher raw material costs, it was decided to use a chopped strand glass fibre mat material with randomly oriented reinforcement instead as the main objective was demonstrating the sensor technologies.

**Figure 4:**
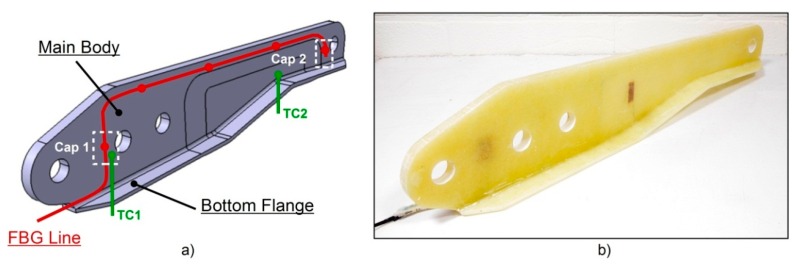
3D concept drawing (**a**) and real component with embedded sensors (**b**) for the A380 hinge arm.

**Figure 5:**
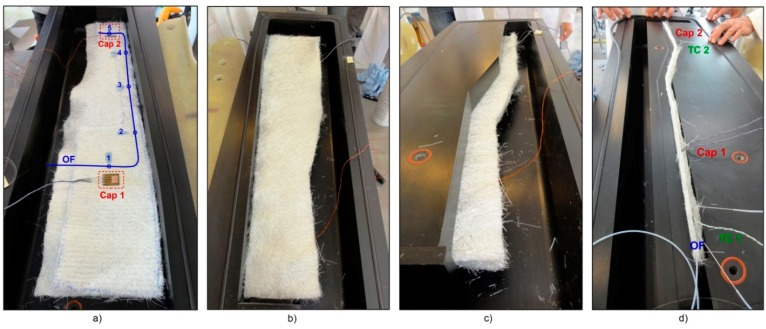
Preform preparation and sensors embedding step sequence for the A380 hinge arm. Sensors layout (**a**); preform preparation (**b**); positioning of the top mould insert (**c**); positioning of the bottom flange insert (**d**).

The component consisted of a main body and a bottom flange at its base as can be seen from Figure 4. The main body had four holes which were drilled after production where the droop nose mechanism actuators could connect. In addition, the component needed also a final contour trimming to meet its design dimensions. A total of three layers of chopped strand mat were cut to size and placed into the aluminium mould. Each of these layers was 6 mm thick, for an overall thickness after curing of 17.5 mm. An optical fibre line (OF) carrying five FBG sensors was placed between the second and third mat layer, as indicated in Figure 4a. Next to FBG_1_ and FBG_5_, two planar inter-digitated capacitive sensors were positioned: more in detail, next to FBG_1_ an in-house developed sensor 68 μm thick and with a sensitive area of 12 × 15 mm^2^ was used. Instead, next to FBG_5_ an IDEX115 sensor from Netzsch^®^ having a thickness of 190 μm and a sensitive area of 25 × 10 mm^2^ was used. FBG_2_, FBG_3_ and FBG_4_ were laid in a straight line going from the resin inlet towards the resin outlet, in order to be able to follow the resin flow front during injection. In order to have information on the temperature development during the production process, two type K thermocouples—TC1 and TC2 indicated in Figure 4a—were also embedded close to the capacitive sensors. Once that the third layer was correctly positioned, the top mould insert was fixed in place as it can be seen from Figure 5c. The fibre glass part exceeding the insert was bent 90° upwards for the top layer and 90° downwards for the bottom layer. Accordingly, the sensor cables were also bent 90° upwards and a last layer of glass fibre mat was positioned vertically to form the bottom flange. A second insert was positioned against these vertical plies—Figure 5d—and a third wedged insert was used in order to fill the mould cavity. The sensor cables were housed in a small resin channel (approx. 1.7 × 15 mm^2^) running along the mould top edge and forced through the mould resin outlet. The mould design allowed the cables to exit via an already existing extra-hole, which was properly sealed with some silicone to avoid any resin leakage during production, as well as to avoid any vacuum breaches which could cause voids in the part. Lastly, the upper mould lid was fixed and the mould sealed.

## 6. Results and Discussion

In this section the results of the production monitoring for the A380 hinge arm will be introduced. The RTM facilities used for its production were described in Section 4.1. The resin chosen for the process was a two-component epoxy system from Huntsman^®^ (The Woodlands, TX, USA), namely Araldite LY 1564 resin and Aradur XB 3486 hardener. The manufacturer’s datasheet indicated a curing cycle of 48 h at 23 °C, which is reduced to 20 h when the temperature is increased to 40 °C. In order to improve the resin’s mechanical properties, a post-curing cycle is suggested. The two components were separately preheated at 60 °C, mixed with a weight ratio of 100:34 and injected at 5 bars pressure into the mould through the inlet channel. Vacuum was applied to the mould, in order to evacuate the preform of possibly trapped air bubbles. The whole injection phase took about 10 min. Once the resin reached the outlet tubing, the pump was disconnected and the inlet and outlet tubing clamped. The whole RTM curing process lasted about 28 h. Afterwards, the component was demoulded and post-cured in an oven for 8 h at 80 °C.

### 6.1. Cure Monitoring

**Figure 6:**
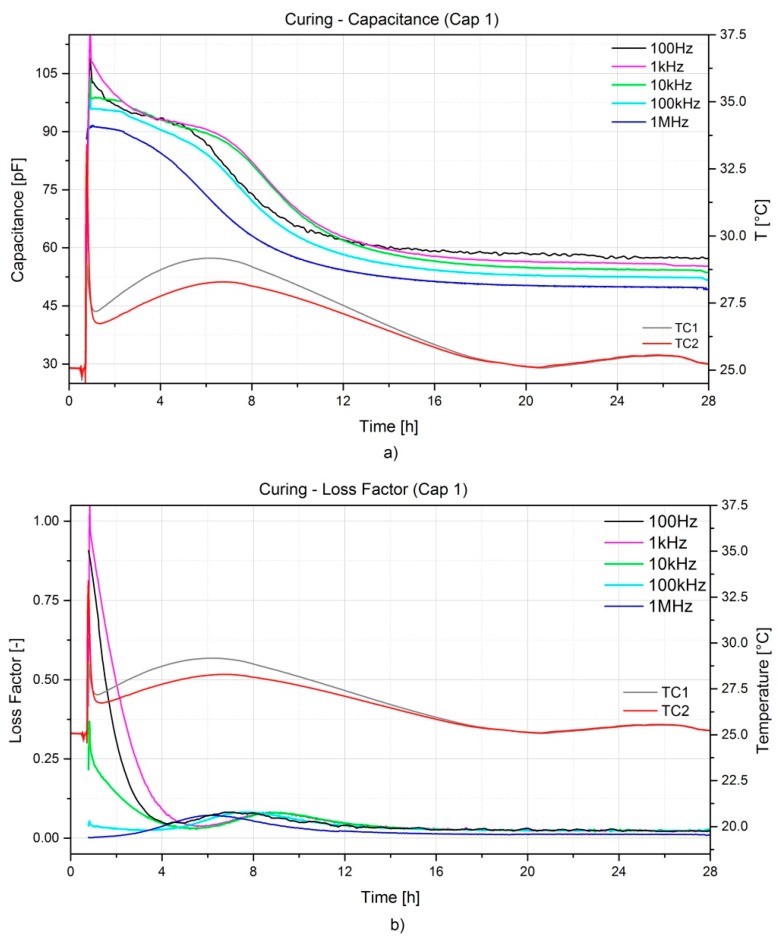
Capacitance (**a**) and loss factor (**b**) evolution during the RTM process of the A380 hinge arm.

**Figure 7:**
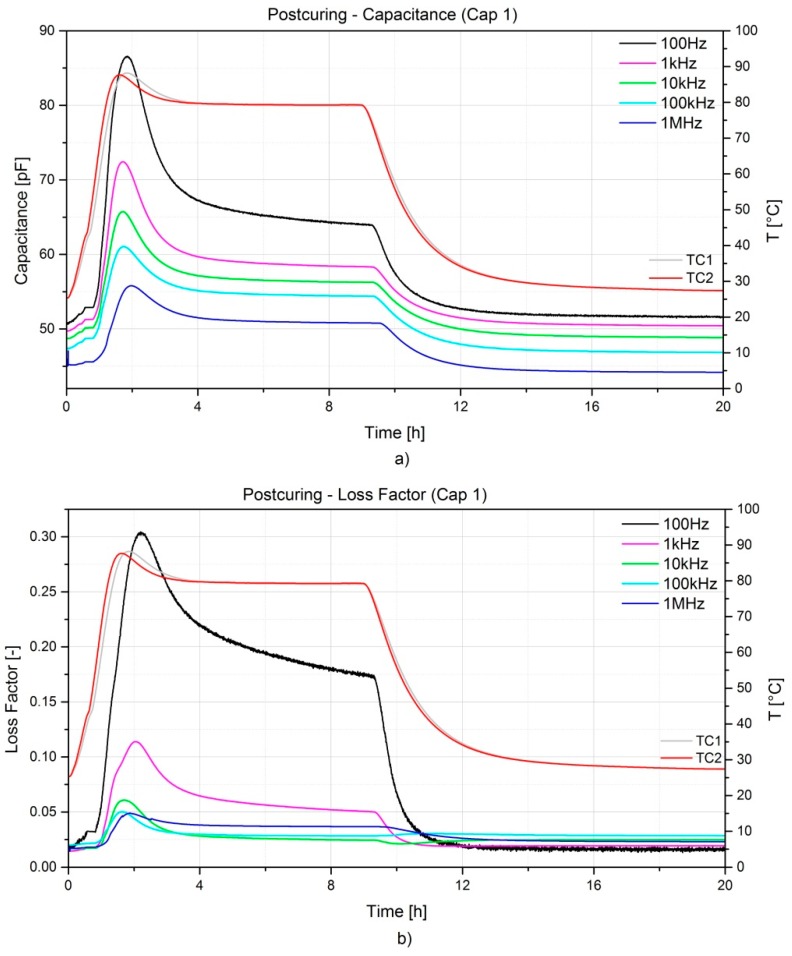
Capacitance (**a**) and dissipation factor (**b**) evolution during the post-curing at 80 °C for 8 h in the oven for the A380 hinge arm.

This section will introduce the results related to the data acquired with the two capacitive sensors embedded at the two different locations in the A380 hinge arm, namely close to the inlet (Cap 2) and close to the outlet (Cap 1) of the RTM mould. As described in Section 4.3, the two sensors were connected to two different pieces of equipment. The measurements were acquired at different frequencies ranging from 100 Hz to 1 MHz. The most relevant curves for the capacitance and the loss factor during the curing and the oven post-curing are presented in Figure 6 and Figure 7, respectively. During the curing process two distinct phenomena are occurring. As the resin starts off in a liquid state, one has the free ions moving into the media and the movement of the dipoles. As the crosslinking reaction evolves, the network gets stronger inhibiting the dipole movements. The capacitance and the loss factor decrease accordingly. The curing process can be considered completed when both capacitance and loss factor reach their asymptote.

The frequency dependency on the measurements is remarkable; generally, lower frequencies show higher sensitivity, but are more influenced by noise. On the contrary, higher frequencies show a lower variation of the measured magnitudes. Depending on the resin system and its curing kinetics, an optimal frequency can be selected. In the case presented the optimal frequency was found at 10 kHz, as a good trade-off between noise and sensitivity. Moreover, measurements are also temperature dependent as it can be noticed from Figure 7 during the oven post-curing. Shortly after the heating step (1 h), a considerable increase of both capacitance and dissipation factor is noticeable and this is directly connected to the amount of latent curing which is activated during the heating. This peak levels off to a constant value which reflects the pure influence of temperature on the measurements. In addition, the peak exhibits a shift in time when higher frequencies are considered. The final values of both capacitance and dissipation factor after cooling down are comparable with the initial ones, lower frequencies show slightly higher values with respect to the initial, while higher frequencies show slightly lower values. The noise influence on the measurements was rather limited for the post-curing if compared to the curing process; this can be related with the ion diffusion and the dipole oscillation becoming limited as the resin solidifies.

**Figure 8:**
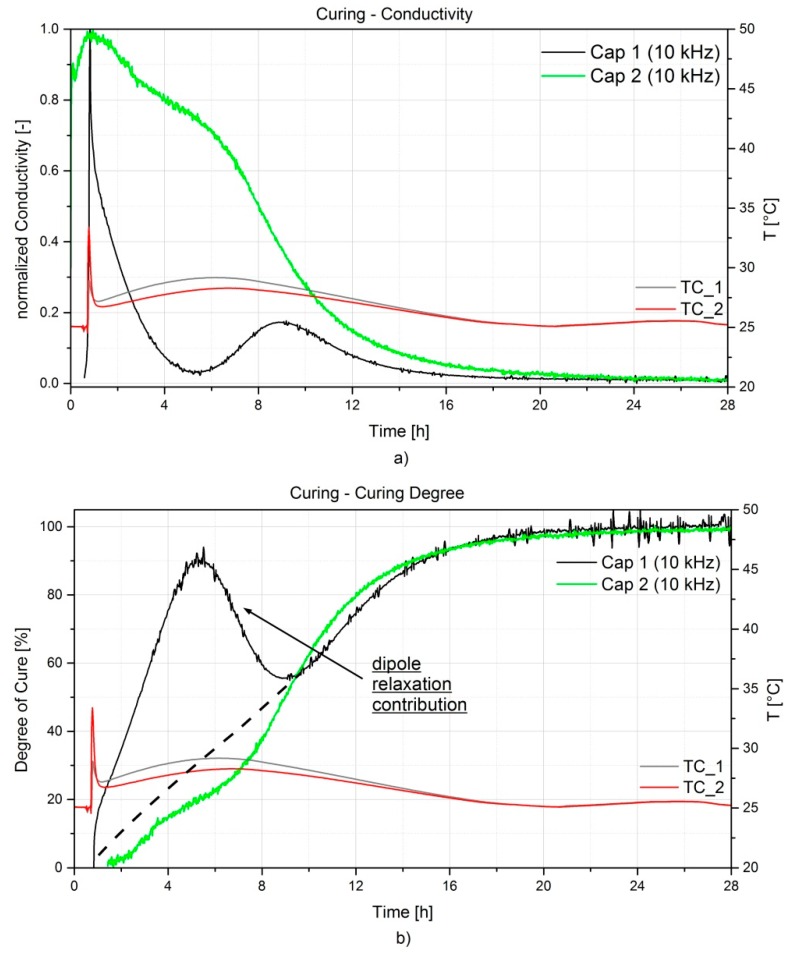
Evolution of the normalized ion conductivity (**a**) and of the degree of cure in % (**b**) for the two capacitive sensors during the RTM curing process of the A380 hinge arm.

The ion conductivity σ [S/cm] is often used to define the curing behavior of a resin system, since it describes better its changes in physical properties (*i.e.*, viscosity, degree of cure). As mentioned in Section 3, the step from the loss factor to the ion conductivity can only be made under the condition that the contribution of the dipole relaxation to the loss factor is negligible (*i.e.*, Equation (11)). This is verified when the ion conductivity curves overlap (no frequency influence) at least in the early curing stage. The selection of the optimal frequency is of importance to define a meaningful curing profile curve. Low frequencies are preferable at early curing stage, since typical dipole relaxation time is low, but low frequencies are more susceptible to electrode polarization effects occurring when the ion mobility is high. The ideal frequency should not be influenced from both dipole relaxation and electrode polarization [34,35]. Furthermore, as the curing evolves the ion mobility and dipole movements become restricted and therefore higher frequencies describe better the end-of-curing behavior [13]. For this resin system, the optimal frequency was selected at 10 kHz. The conductivity was derived from the dissipation factor according to Equation (11) and normalized. The resulting curves of the two sensors are depicted in Figure 8a. As one can notice, a difference between the curve trends exists, even when the same frequency was considered. This is highlighted also in Figure 8b, Cap 1 (*i.e.*, LCR meter measurements) where a sudden increase of the degree of cure is followed by an unexpected decrease, which does not have a physical meaning. This might be due to the contribution given by the dipole losses $\varepsilon_{dipole}^{\prime \prime }$ at the early stage of curing, which needs to be subtracted when defining the degree of cure. Some resin systems can even contain a high level of ionic particles (additives or impurities), which will gather at the sensor electrodes. This will result in a much higher initial conductivity value. As a consequence, any dipolar loss peaks in the first stage of curing can be masked and left completely unnoticed. The same can occur if the sensor design is susceptible to ion polarization disturbances. On the other hand, an accurate read-out apparatus and a good sensor design can allow for a more precise measurement of the capacitance and dissipation factor. This will highlight, if present, any peak related to the dipole relaxation as it is seen on the black curve in Figure 8b related to Cap 1, which exhibits a peak at 5 h from the beginning of the curing. This however does not affect the later stage of the curing, since the increased cross-linking limits the dipoles movement and the contribution of the ion conductivity $\varepsilon_{ion}^{\prime \prime }$ prevails. In fact, approximately 9 h after the resin injection, the two curves begin overlapping. After approximately 20 h from the injection, one can observe a cure degree of about 98% and not further increase has been captured afterwards. It is important to mention that the 100% degree of cure has been defined for a 30 h curing cycle at room temperature, but during the following post-curing some additional reaction has been noticed, as already reported in Figure 7.

### 6.2. Residual Strains

The production monitoring results from the fibre optic sensors will be disclosed in this section. Spectra, as well as wavelength peak shifts were acquired throughout the RTM curing and the oven post-curing. The initial (*i.e.*, before embedding) and the final (*i.e.*, after post-curing) wavelength peak values λ*_B_* on OF are listed in Table 1.

**Figure 9:**
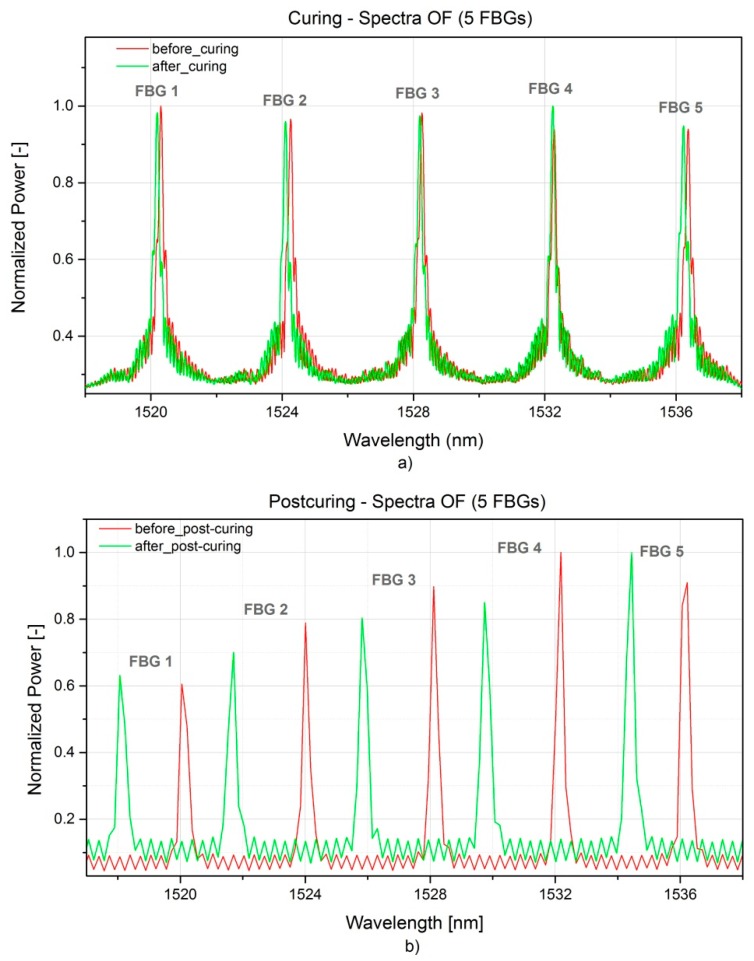
FBG spectra acquired during RTM production (**a**) and post-curing (**b**) of the A380 hinge arm.

In Figure 9a,b the full spectra acquired before and after RTM production and before and after post-curing are presented. For clarity, the spectra referring to the curing were acquired once that the mould was closed, so prior to the injection, and after 30 h curing before the demoulding. No peak distortion was observed, but in general a lower peak reflection could be seen after post-curing. This has nothing to do with a physical effect on the embedded sensors, but most probably it is related to a non-optimal selection of the integration time for the spectra on the interrogator acquisition software. It is worth to mention that in spite of this, the accuracy of the measurements is not compromised.

**Figure 10:**
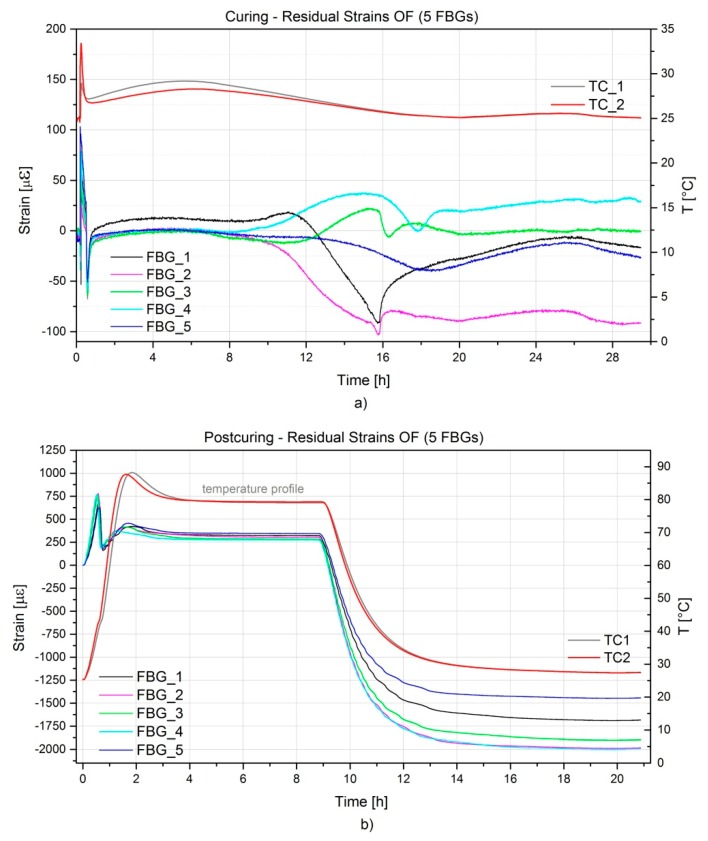
Residual strains evolution during RTM production (**a**) and post-curing (**b**) of the A380 hinge arm.

In Figure 10a,b strain development, defined from Equation (6) as described in Section 2.3 during the curing and post-curing process is represented. As previously mentioned, the two-component resin was preheated at 60 °C prior to the infusion. By the time it reached the first thermocouple the resin had already cooled down considerably to 33 °C, and when it reached the second one the temperature reading was 29 °C. The first FBG sensor reached by the resin was FBG5 as noticed also by an increase of the temperature on TC2. Finally, FBG1 was wet and after about 10 min the resin reached the outlet tubing. Aside from the observed initial wavelength peak shift due to the resin flowing over the FBG sensors, the long cure at room temperature does not affect the sensors’ spectra and the influence on the wavelength shift is negligible. Nevertheless, one can notice the vitrification stage (starting at 9 h from the injection phase) accompanied by a resin chemical shrinkage, the latter inducing on FBG_1_ and FBG_2_ compressive strains up to approx. 100 με. At the end of the vitrification (about 16 h from the injection) the component starts detaching from the mould and, as a consequence, a decrement of the strains leading to a neutral condition is noticed. Only for FBG_2_ the compressive strain is maintained. This could be related to the specific embedding location and to a local variation of the fibre volume fraction. The vitrification is also visible in Figure 6a, when the capacitance starts to decrease drastically at about 6 h after the injection until about 16 h when the curing becomes limited as a consequence of the reached glassy state.

After the post-curing in the oven at 80 °C, a considerable shift in the wavelength of the reflected spectra is observed. Analyzing Figure 10b, one can observe during the heating-up that thermal strains are initially induced on the component, but at approximately 37 °C a release of strains (from 750 με to 250 με) is seen. This temperature corresponds to the *T_g_* of the resin system after curing at room temperature, as stated from the resin technical datasheet (33–37 °C) [36]. It follows a slower increase of strain to a peak value, which is indicating that some additional reaction is happening in the resin (also noticeable from the increase of temperature). Afterwards, the strain is maintained constant and upon cooling down to 27 °C a compression strain up to −2000 με is seen. It is to be noticed that FBG_1_ and FBG_5_ see a lower compression strain (namely −1450 με and −1680 με) compared to the other sensors. This could be related to their orientation with respect to the other sensors. Additional tests were performed in order to define the thermal expansion coefficients on the hinge arm at two different FBG locations by means of surface mounted strain gauges. FBG_1_ and FBG_4_ were selected. The experiments showed that a similar CTE value of 40 × 10^−6^ K^−1^ was measured for the two differently oriented strain gauges. Such CTE value would explain the amount of residual strain measured during cooling down to room temperature in the post-curing phase. However, this could not explain the different values of residual strains measured on the different FBGs. It is the authors’ belief that local fibre distribution and/or richer resin content might influence the amount of residual strain measured. It is worth to mention that a next temperature cycle up to 70 °C was performed on the hinge arm and strains were acquired by means of the embedded FBGs. No additional residual strains were generated and the only effect was given by the thermal strain. This is in accordance with the resin technical datasheet which set the new *T_g_* after post-curing to 80–84 °C and therefore heating to 70 °C would not induce any change in the material.

## 7. Conclusions

This work has proved the feasibility of successfully embedding a sensor network based on FBGs, capacitive sensors and thermocouples in an aerospace composite component produced by RTM. This has been achieved without requiring any changes to the mould geometry or to the component design. The production process was monitored throughout the RTM injection and curing phases. The component was successfully demoulded without harming the sensors and post-cured in an oven, where measurements were also acquired. The FBGs have proved being capable of detecting the increase of temperature and of pressure inside the mould as well as the resin filling. During the post-curing process, the FBG sensors were useful to evaluate the amount of residual strains build-up due to thermal expansion. Values up to −2000 με have been measured upon cooling after post-curing on the A380 hinge arm. This is to be expected since the fibre volume fraction on the preform was kept intentionally low in order to have higher resin content. Therefore the shrinkage is mainly dominated by the resin thermal expansion. Moreover, the capacitive sensors have been capable of successfully following up the curing and post-curing cycle on the A380 hinge arm. Finally, a curing degree profile has been presented for both embedded sensors. This proved to be an *in situ* measuring technique with a lot of potential for further development. After production, the FBG sensor network was used to perform dynamic measurements in order to detect the first eigenfrequencies of the component and compare them with the one obtained from a well-established Laser Doppler Vibrometer modal analysis technique. The results are disclosed in the work of Lamberti *et al.* [37].

## Figures and Tables

**Table 1:** FBG central wavelengths λ_B_ before and after production for the A380 hinge arm.

Grating	Initial λ_B_ (nm)	Final λ_B_ (nm)
Fbg_1_	1520.279	1518.165
Fbg_2_	1524.255	1521.722
Fbg_3_	1528.245	1525.915
Fbg_4_	1532.262	1529.828
Fbg_5_	1536.320	1534.467
